# Fabrication and Experimental Study of Micro/Sub-Micro Porous Copper Coating for Anti-Icing Application

**DOI:** 10.3390/ma16103774

**Published:** 2023-05-16

**Authors:** Jingxiang Chen, Cheng Fu, Junye Li, Weiyu Tang, Xinglong Gao, Jingzhi Zhang

**Affiliations:** 1 Facility Design and Instrumentation Institute, China Aerodynamics Research and Development Center, Mianyang 621000, China; jingxiangchen@zju.edu.cn (J.C.);; 2 State Key Laboratory of Aerodynamics, China Aerodynamics Research and Development Center, Mianyang 621000, China; 3 Hangzhou Global Scientific and Technological Innovation Center, Zhejiang University, Hangzhou 311215, China; 4 Department of Energy and Power Engineering, Shandong University, Ji’nan 250061, China

**Keywords:** superhydrophobic surface, anti-icing, porous coating, wettability

## Abstract

Micro and sub-micro-spherical copper powder slurries were elaborately prepared to fabricate different types of porous coating surfaces. These surfaces were further treated with low surface energy modification to obtain the superhydrophobic and slippery capacity. The surface wettability and chemical component were measured. The results showed that both the micro and sub-micro porous coating layer greatly increased the water-repellence capability of the substrate compared with the bare copper plate. Notably, the PFDTES-fluorinated coating surfaces yielded superhydrophobic ability against water under 0 °C with a contact angle of ~150° and a contact angle of hysteresis of ~7°. The contact angle results showed that the water repellency of the coating surface deteriorated with decreasing temperature from 10 °C to −20 °C, and the reason was probably recognized as the vapor condensation in the sub-cooled porous layer. The anti-icing test showed that the ice adhesion strengths of the micro and sub-micro-coated surfaces were 38.5 kPa and 30.2 kPa, producing a 62.8% and 72.7% decrease compared to the bare plate. The PFDTES-fluorinated and slippery liquid-infused porous coating surfaces both produced ultra-low ice adhesion strengths of 11.5–15.7 kPa compared with the other non-treated surfaces, which showed prominent properties for anti-icing and deicing requirement of the metallic surface.

## 1. Introduction

Excessive ice accumulation on the surface always results in a variety of critical issues and even undesired catastrophic problems, for example, transmission line malfunction, excessive energy consumption, and aircraft crash accident. Superhydrophobic coating with micro/nanoscale structures, especially for the spherical particle coatings, can obviously reduce the contact area between the liquid drop and the substrate due to the air existence and the curvature of the particles in the porous structures. The precise control of the particle size and surface structure guaranteed fine-tuning of the coating porosity and superhydrophobic characteristics, such as the surface wettability [1,2,3,4,5], surface thermal conductivity [6,7,8,9,10,11], diversity types of ice accumulation [12,13,14,15], ice adhesion [16,17,18,19] and droplet impacting dynamics on different superhydrophobic surfaces [20,21,22,23].

Most of the pioneering studies were focused on presenting and validating the superhydrophobicity and anti/deicing characteristics of different textured, coated, or chemical-induced surfaces, especially for the superhydrophobic surface with micro/nanostructures. Young’s equation [24] revealed the equilibrium behavior of a pure water drop on an ideal smooth substrate. Subsequently, Wenzel [25] and Cassie–Baxter [26] equations were proposed to explain the contact model and transition model between water drop and rough surface. Plenty of types of superhydrophobic surfaces reported in current literature were fabricated principally based on this mechanism, which showed excellent water repellency and promising capability of anti-icing application. The superhydrophobicity of the periodic textured surface produced by laser cutting or template method is subjected to the geometric parameters of the micro/nanostructure. Zhu et al. [27] found that the micropillar distance of the substrate rough layer played a decisive role in water repellency and adhesion force, indicating a smaller micropillar distance contributing to reducing adhesion force. Li et al. [28] simulated the nanodroplet impacting on the stripe-textured surfaces, and they reported that the textured structures redistributed the forces acting on the droplet resulting in anisotropy behavior and less contact time. A combination of micro textured structures and irregular nano surface roughness can significantly increase the contact angle as well as reduce the contact time and contact area. Experimental results of Sarshar et al. [29] indicated that hierarchical structures were more effective in reducing the contact angle and length of the three-phase contact line, both of which were key parameters affecting the ice formation speed to sustain the Cassie-Baxter state. They also pointed out that superhydrophobic surfaces with higher anti-icing efficacy may fail to produce excellent deicing performance due to the different icing mechanisms and surface structures.

The icing on a subcooled surface is more complicated than observed, while it is commonly accepted that ice is generated on a cold substrate surface because of the freezing of water adsorbed or condensed on the surface structures below the freezing point. Farhadi et al. [30] examined the icing/deicing performance of several nanostructured superhydrophobic coatings, and they found that anti-icing performance significantly deteriorated with test circles. They also implied that surfaces with nanostructures might be limited in highly humid environments due to nano water droplet condensing in the rough structures. This invalidation of anti-icing performance has resulted from the wetted surface on which the water molecules groups and condensed nano water drops are absorbed. Cheng et al. [31] developed a superhydrophobic surface by assembling soft and stiff particles on a glass substrate which showed excellent anti-icing performance, icephobic performance with ultralow ice adhesion, self-deicing ability, and good icing/deicing cycle stability. Rajiv et al. [32] proposed an economically workable method to prepare carbon-based (multiwalled carbon nanotubes and carbon nanofibers) superhydrophobic coatings for long-term-durable anti-icing applications that exhibited a water contact angle of 171.6°. Wang et al. [33] studied the dynamic anti-icing characteristics of water drops impinging superhydrophobic surfaces. Kinetic behaviors of droplets, such as spreading, retracting, rebounding, and splashing, were comprehensively studied. The results showed that the surface temperature significantly influenced fully rebounding behavior, which determined the temperature limit of superhydrophobic surfaces for anti-icing applications. Additionally, Yang et al. [21], Zigmond et al. [22], and Natsui et al. [34] also studied the dynamic behavior of water droplet icing on superhydrophobic surfaces, and they discovered that surficial chemical substances also had noticeable effects on droplet icing behaviors.

For designing a better anti-icing surface, Jung et al. [35] pointed out that the competing influence of wettability and surface roughness must be well optimized, which indicated that a super water-repellent surface might not produce the expected anti-icing performance. In addition to this, the content of low surface energy chemicals in porous surfaces is also related to surface roughness and porosity. Low surface energy chemicals, such as fluoroalkyl silane and polydimethylsiloxane (PDMS), were commonly used to prevent hydrogen bond formation from decreasing adsorption force between the ice layer and substrate. Jiang et al. [36] investigated condensation frosting on four typical surfaces, and they found that the shear stress force of the slippery surface was highlighted by orders of magnitude lower than that on the bare surface. Their MD simulations also imply that the traditional water-solid contact mode can change into water-oil-solid contact mode, which prevents icing propagation. Kreder et al. [37] reviewed massive works on fabricating superhydrophobic surface (SHS) and passive anti-icing strategies, and they found out that there was no single surface that had shown the ability to rapidly shed impact and condense water droplets, suppress ice nucleation, and reduce ice adhesion, all while operating in various environments with high durability and longevity. From this standpoint, deicing and anti-icing issues are still not perfectly solved, which will be a challenging and attractive topic for a long time.

In this work, we proposed a copper powder sintered porous coating surface to transform hydrophilic copper substrate into a superhydrophobic surface and further introduced 1H,1H,2H,2H-Perfluorodecyltriethoxysilane (PFDTES) to decrease the surface energy of the coating layer. This type of coating surface has comparable mechanical strength with the substrate material and can be further developed as a slippery liquid-infused porous coating surface to reduce ice adhesion strength. The lubricating material can be firmly absorbed in the capillary channels by utilizing the capillary effect compared with other textured surfaces. The diameter of copper powder, the sintering process, and the raw material ratio were experimentally tested to fabricate an ideal anti-icing functional surface. The porosity was well designed to maintain a metastable water layer in the porous channels to suppress ice nucleation. In this paper, two types of micro and sub-micro coating surfaces were fabricated, and the anti-icing mechanism was discussed in detail. The anti-icing performance of the porous coating surface and its derived surface, including the superhydrophobic surface fluorinated with PFDTES and the slippery liquid-infused porous surface with simethicone, were also discussed in the following sections.

## 2. Experimental Methodology

### 2.1. Materials

Two types of micro/sub-micro copper powder with an average diameter of ~30 μm and ~500 nm were purchased from (Shanghai Chaowei, Inc., Shanghai, China) to prepare different porous copper coatings. 1H,1H,2H,2H-Perfluorodecyltriethoxysilane (PFDTES, CAS No.101947-16-4), Hydroxypropyl Methylcellulose (HPMC, CAS No.9004-65-3), zinc stearate, ethanol, ethanediol, and 98% wt. sulfuric acid were purchased from Aladdin (Delaware, IA, USA). Deionized water, 17% wt. hydrochloric acid, simethicone, and acetone were supplied by Macklin Inc. (Shanghai, China). All chemical materials were of analytical purity and were directly utilized without any further synthetic product and purification.

### 2.2. Slurry Preparation

**Figure 1:**
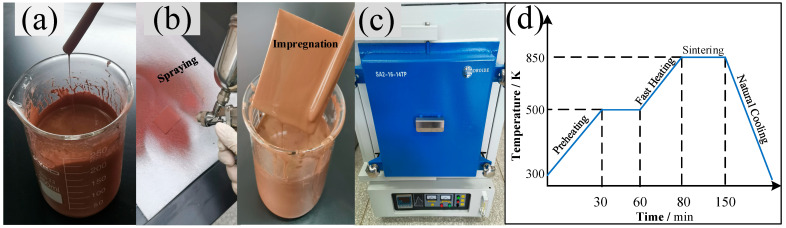
Experimental shot cuts for the preparation of the sintered porous copper coating surface. (**a**) the preparation of the Cu-powder/HPMC/ethanol slurry; (**b**) the spraying and impregnating method for the preparation of the initial coating layer on a bare copper substrate; (**c**) the high-temperature furnace with reducing atmosphere used to produce the porous coating surface; (**d**) the temperature control curve used for fabricating the copper powder porous coating.

The copper powder slurry is a homogeneous mixture that consists of microspherical copper particles, HPMC binder, solvent, and a small amount of dispersing agent. First, the binder colloid was prepared by mixing 2.0 g HPMC with 100.0 mL hot water at a temperature of ~70.0 °C because the HPMC was difficult to dissolve in water at room temperature. Secondly, the dispersing agent was obtained by dissolving 3.5 g zinc stearate powder with 100.0 mL ethanol, and the mixture was heated to ~45.0 °C by a thermal oil bath to make the zinc stearate fully dissolved and dispersed. Subsequently, the Cu-powder/HPMC/ethanol mixture was prepared with the mass ratio of 5/1/4 and 4/1/3 for 30 µm and 500 nm copper particles, respectively. During this process, the zinc-stearate/ethanol solution was added manually by a burette when the Cu-powder/HPMC/ethanol slurry was stirred by a high-speed shear apparatus (YFA-25, FLUKE, Everett, WA, USA) to adjust the viscosity of the slurry to an expected value (the copper particles can homogeneously adhere to the glass rod, as Figure 1a shows). The same preparation method was applied to the preparation of Cu-powder/HPMC/ethanol slurry with different copper particle diameters and weighted powder mass ratios.

### 2.3. Fabrication of Porous Coating

**Figure 2:**
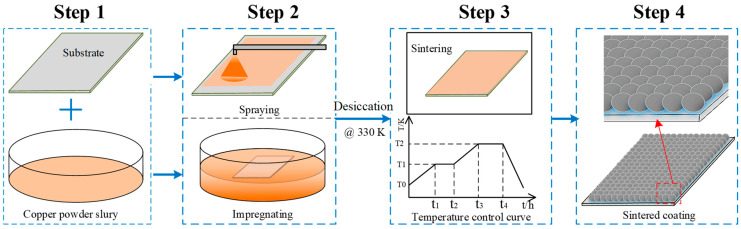
Schematic of preparation of the copper powder sintered porous coating surface.

Two different types of copper powder particles having predetermined Gaussian distributions with mean diameters of ~500 nm and ~30 μm were selected to prepare the Cu-powder/HPMC/ethanol slurry. The size range and geometrical shape of each type of copper powder were also predetermined to get an optimal pore size and roughness structure. The fabrication methods to prepare the porous coating surface were summarized as four steps shown in Figure 2, including the preparation of clear substrate and slurry, spraying or impregnation, volatilizing and sintering in a furnace, and then naturally cooling. The porous coating surface was obtained by spraying the as-prepared slurry on a clean and dry copper plate which was ultrasonically degreased first in acetone solution and cleaned in ethanol for 10 min. As in Figure 1b, another method of directly immersing the polished substrate into the slurry was also tried to make a comparison with the spraying coating. As illustrated in the former section, the solution’s basis material was ethanol; the spraying or immersion surface can be quickly dried in a thermostatic oven at a constant temperature of 60 °C for 2 h. Subsequently, the dried coating plate was transferred to a high-temperature resistance furnace, as shown in Figure 1c. As presented in Figure 1d, a temperature control curve was elaborately designed consisting of five operations, including preheating, thermostatic volatilizing, fast heating, sintering, and the natural cooling process. To avoid carbonization and oxidation under the sintering temperature, the HPMC ingredient in the surface coating was thermostatically volatilized for 30 min at a chamber temperature of 230 °C. Meanwhile, the vapor steam was driven out by continuous hydrogen flow and combusted at an outlet (10 m away from the furnace). Subsequently, the temperature of the furnace chamber was rapidly increased to the sintering point, *T* = ~0.85 ± 0.05 *T_melt_* (different temperatures for different particle diameters). Temperatures of 1153 K and 1205 K were validated for ~500 nm and ~30 µm copper particles in this experiment. Under this condition, the copper particle maintained a semi-molten state and adsorbed on the substrate. Finally, the porous coating was formed during the sintering process under a reducing atmosphere, and the sintering template was naturally cooled by directly shutting down the power supply.

### 2.4. Surface Modification

**Figure 3:**
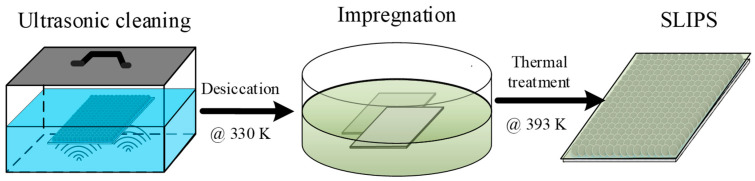
Preparation of the slippery liquid-infused porous coating surface by further utilizing the as-prepared porous coating surface. The coating surface was cleaned in acetone solution, then transferred into the PFDTES/ethanol solution. The SLIPS surface was obtained by removing the excess oil.

To obtain the superhydrophobic and anti-icing surface, the as-prepared porous coating surfaces needed further surface treatment, including low surface energy treatment and surface lubrication. Although the micro/sub-micro roughness enhanced the water repellency in contrast to the bare surface, the free energy of metal was still too high to achieve a superhydrophobic state—a low surface energy solution composed of 2.0% wt. 1H,1H,2H,2H-Perfluorodecyltriethoxysilane (PFDTES)/ethanol solution was prepared. Figure 3 shows that all the as-prepared porous coating surfaces were firstly ultrasonic cleaned in acetone solution and immersed in PFDTES/ethanol solution for 2 h to reduce the surface energy. Secondly, the coating surfaces were transferred into a thermostatic drier box and were thermally treated at 120 °C for 60 min to obtain the PFDTES-grafted superhydrophobic surface (SHS). This annealing process facilitates the evaporation of remaining solvents, and the thermal energy can make the atoms and polymeric group activated to form stronger bonds. For anti-icing considerations, a slippery liquid-infused surface was also prepared by injecting simethicone into the porous coating layer, and the excess oil was removed by vertically placing the surface plate for 24 h.

### 2.5. Characterization

**Figure 4:**
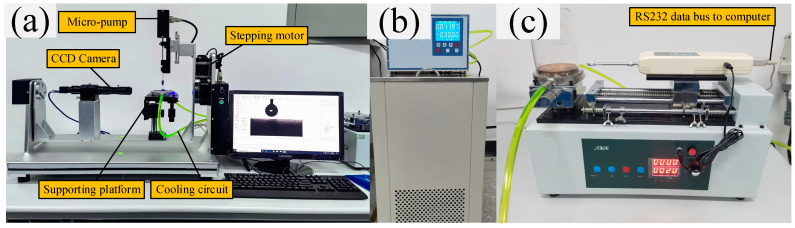
Experimental devices of (**a**) surface wettability measurement, (**b**) low-temperature thermostat with the working fluid of ethylene glycol, and (**c**) pull-push force test platform for ice adhesion strength measurement.

The microstructure of the coating surface was measured by field emission scanning electron microscopy, and the surface component of chemical elements was analyzed by the energy-dispersive X-ray spectroscopy method. The surface wettability, including contact angle, receding angle, advancing angle, and contact angle hysteresis, was measured by a contact angle meter at different surface temperatures, as displayed in Figure 4a. Figure 4b shows that a low-temperature thermostat was used to change the temperature of the substrate surface (the achievable temperature range is from room temperature to −40 °C). The volume of the water droplet was controlled within 5 ± 0.1 μL by a step micro-pump. Six random positions of each coating surface were selected during the wettability characterization, and the mean angle value was calculated as the test result.

A hollow cylinder with an inner diameter of 20 mm made from glass and with low surface energy treatment was placed on the test surface to prepare the icicle. Firstly, 2 mL portion of deionized water was added into the hollow cylinder and frozen for 10 min under the environment at −20 °C. Secondly, the remaining 4 mL portion of deionized water was again added and frozen for 30 min at the same cooling temperature. Finally, the adhesion force stress between each coating surface and the icicle surface was measured by a pull-push dynamometer clamping (with an accuracy of ±0.01 N) on the automatic test platform, as Figure 4c displayed. An effective heat-insulating box was designed to prevent convective heat transfer and water condensation from the atmosphere to the substrate plate during the anti-icing test. The low-temperature thermostat was also used to change the icing and surface temperature during the wettability experiment. All data were recorded by data acquisition software and restored for the subsequent data analysis.

## 3. Results and Discussion

### 3.1. Surface Topography

**Figure 5:**
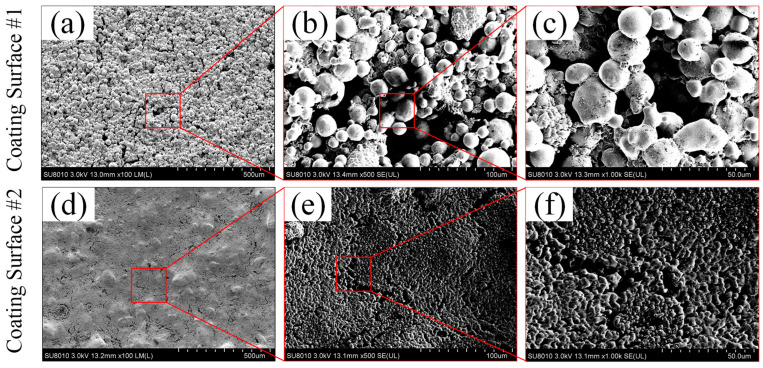
Surface topography and partial enlarged view captured by a field emission scanning electron microscopy (FE-SEM) in terms of (**a**–**c**) for the coating surface CS#1 and (**d**–**f**) for the coating surface CS#2, with a magnification factor of 100, 500 and 1000, respectively.

The surface morphologies of the two sintered porous coating surfaces were measured by field emission scanning electron microscopy (FE-SEM). As shown in Figure 5, ~30 µm and 500 nm copper particles were used for preparing the coating surface CS#1 and CS#2, respectively. The spherical copper powder particles were welded firmly with each other on the as-prepared coating surfaces (CS#1 and CS#2). Micro and sub-micro gaps between particles were formed, and most of the particle gaps were connected in the porous coating layer. Compared with the coating surface CS#1 (coated with ~30 µm copper particles) shown in Figure 5a–c, sub-micro-gaps and cavities formed in the coating layer CS#2 were much more intensive, and some copper particles were overheated and melted at the same sintering temperature, which resulted in the destruction of the original spherical morphology. It should be noted that the surface roughness amplitude was determined by the difference of particle stacks in the surface normal direction, and the roughness structure was randomly distributed. The curvature of the surface roughness and the cavity size were fully dependent on the particle diameters, as shown in Figure 5c,f. These two types of sintered coating surfaces were further modified under the usage of 2% wt. PFDTES/ethanol solution and silicone oil to reduce the surface-free energy and ice adhesion strength.

### 3.2. Chemical Composition

**Figure 6:**
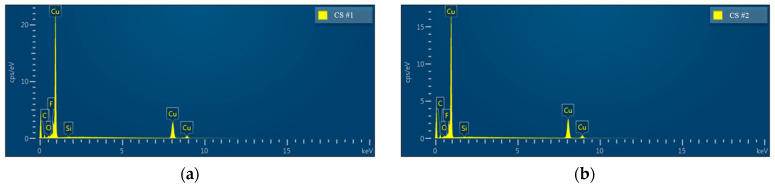
Energy dispersive X-ray spectroscopy (EDS) analysis of the surface chemical element of (**a**) CS#1-PFDTES surface and (**b**) CS#2-PFDTES surface.

The chemical composition of the modified porous coating surfaces (CS#1-PFDTES and CS#2-PFDTES) was investigated by using energy-dispersive X-ray spectrometry (EDS), as shown in Figure 6. Copper, a small number of unreduced bivalent copper ions, and cuprous ions were detected both for CS#1-PFDTES and CS#2-PFDTES surfaces, which indicated that lesser copper oxide and cuprous oxide remained on the copper surface—low surface energy treatment using 2% wt. PFDTES/ethanol solution did not change the surface roughness structure, and in fact, a polymer layer of PFDTES was grafted on the porous surface during the thermalization process at 120 °C. Results of energy dispersive spectrum analysis of the CS#1-PFDTES and the CS#2-PFDTES surfaces are summarized in Table 1. Fluorine was detected for both the CS#1-PFDTES and CS#2-PFDTES surfaces with a mass fraction of 0.75% and 0.49%, respectively. The information of atomic quantity statistics showed that the ratio of fluorine atoms on the surface CS#1-PFDTES was 1.83% and 1.21% for the CS#2-PFDTES surface. The difference in fluorine content between the CS#1-PFDTES and the CS#2-PFDTES surface was because smaller copper particles can be sintered into a lower porosity coating layer, which indicated that more copper atom existed in the same detected region and relatively decreased the number of other atoms. The chemical composition results indicated that these two plates were successfully fluorinated, and a polymer layer with low surface energy was successfully grafted.

### 3.3. Wettability under Different Temperatures

**Figure 7:**
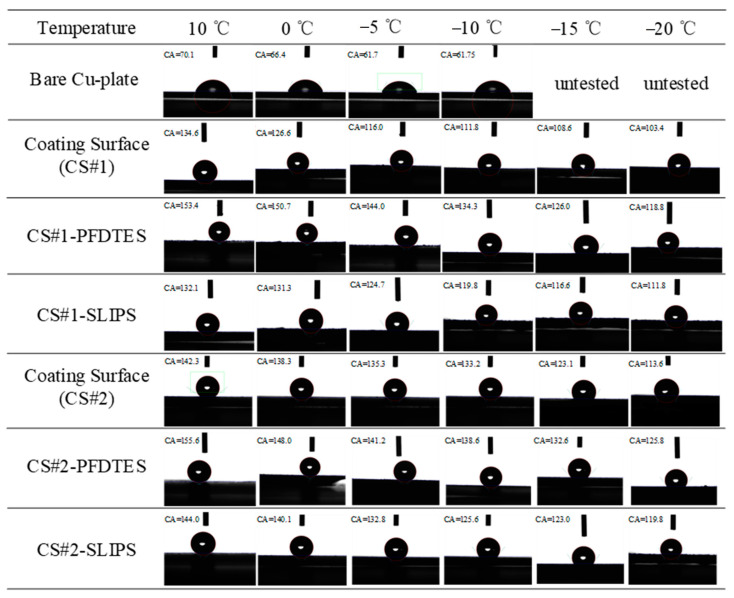
Typical images of static contact angle measurement of 5 μL water droplet on the bare copper plate, porous coating surfaces of CS#1 and CS#2, superhydrophobic porous coating surfaces of CS#1-PFDTES and CS#2-PFDTES, and slippery liquid infused porous surfaces of CS#1-SLIPS and CS#2-SLIPS.

Figure 7 summarizes the wettability characteristics of six porous coating surfaces with different surface modifications that were measured and compared with a bare copper plate. Apparent contact angles (APCA) of the non-fluorinated coating surfaces (CS#1 and CS#2), the fluorinated and slippery liquid-infused porous coating surfaces (CS#1-PFDTES, CS#1-SLIPS, CS#2-PFDTES, and CS#2-SLIPS) were measured by a contact angle meter. A 5 μL deionized water droplet was used for all tests, and the humidity of the environment was not changed (with a temperature and humidity recorder reading of 10 °C and 45%). Figure 7 presents some of the typical images of each contact angle test for different surfaces. The bare copper plate was introduced to make a comparison with the other surfaces, but the contact angles under −10 °C were hard to obtain due to the water droplet suddenly freezing as soon as the water droplet fell on the cold plate. At coolant temperatures of 10 °C, 0 °C, −5 °C, −10 °C, and humidity 45%, the intrinsic contact angle of the used bare copper plate was 70.1 ± 2.48°, 66.4 ± 2.69°, 61.7 ± 2.12°, and 61.74 ± 2.29°, respectively. For the non-fluorinated coating surfaces CS#1 and CS#2 at a coolant temperature of 10 °C, the measured apparent contact angles (ACAs) were 134.6 ± 3.0° and 142.3 ± 2.93°. Under the same test conditions, the measured ACAs of the fluorinated coating surfaces CS#1-PFDTES, CS#2-PFDTES, and slippery liquid-infused surfaces CS#1-SLIPS, CS#2-SLIPS were 153.4 ± 2.64°, 155.6 ± 3.1°, 132 ± 2.94°, and 144 ± 3.23°, respectively.

**Figure 8:**
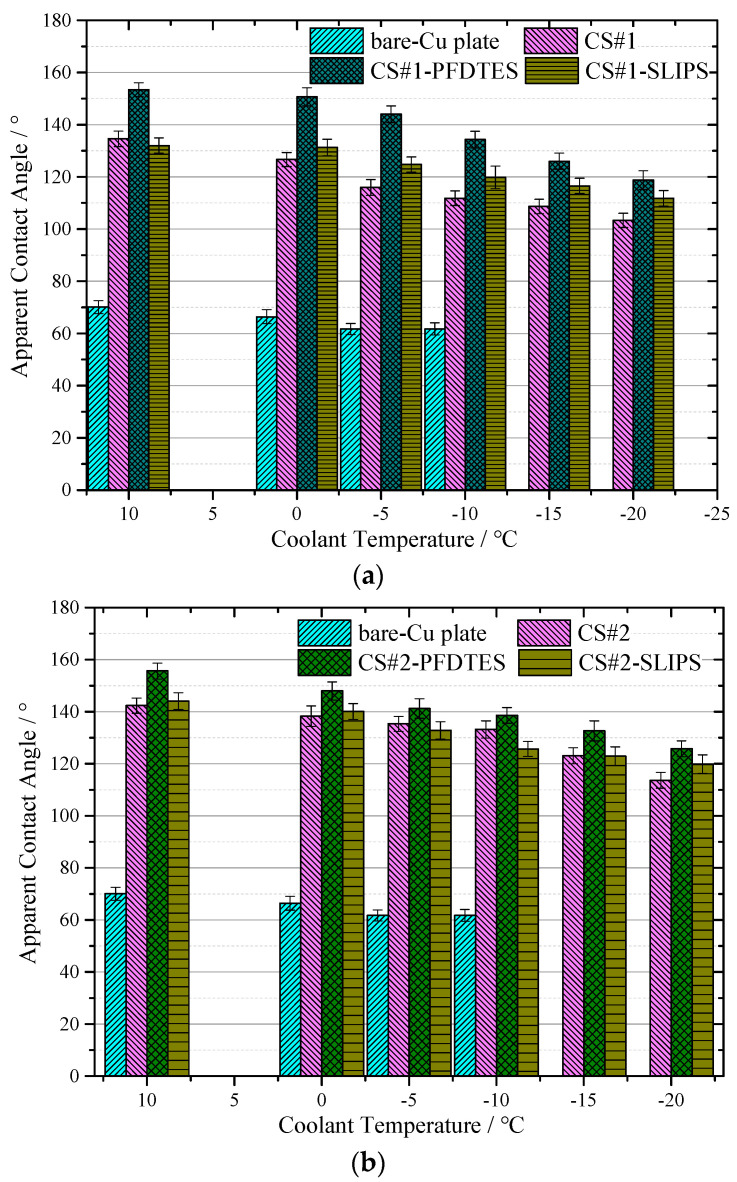
Results of apparent contact angle (**a**) for the coating surface CS#1, CS#1-PFDTES, and CS#1-SLIPS, and (**b**) coating surface CS#2, CS#2-PFDTES and CS#2-SLIPS, measured with coolant temperature at 10 °C, 0 °C, −5 °C, −10 °C, −15 °C and −20 °C, respectively. The contact angle of the bare copper-plated was also measured to make a comparison with all coated surfaces.

Figure 8a,b shows the apparent contact angle near-linearly decreased with reducing the temperature for all the tested coating surfaces, but the capability of water-repellence was much better than the bare copper plate. For the CS#2 series coating surfaces displayed in Figure 8b (with the same particle diameter of ~500 nm), the low surface energy modified surface CS#2-PFDTES yields the best superhydrophobicity with ACA of 155.6 ± 3.1°, compared with the other two surfaces. The surface with a contact angle larger than 150° and contact angle hysteresis less than 10° can be recognized as a superhydrophobic surface [5], on which the Cassie–Baxter contact model is more stable than the other surfaces. The experimental results verified that the water-repellence capability of the superhydrophobic surface decreased with reducing temperature. This was mainly because of the fact that the ACA increased with the increasing surface tension of water, which was inversely proportional to liquid temperature. The scale of roughness structure, *δ* on a superhydrophobic surface, should be significantly smaller than the capillary length [38], *δ* << *l* = (*γ*/*ρg*)^1/2^, which was a principle rule in surface roughness fabrication. Due to the fact that the surface tension *γ* was also inversely proportional to liquid temperature, the roughness effect on contact angle became more important when temperature decreased. Another reason can be the fact that vapor condensed within the porous layer, and the micro/nano water droplet sticky into the porous layer penetrated in the porous layer due to the capillary force, which led to the transition from the Cassie-Baxter contact model to the Wenzel contact model. The condensed water droplets decreased the volume of trapped air in the porous layer and increased the contact area between the water droplet and the substrate, eventually resulting in a decreasing apparent contact angle. The advancing and receding contact angles were also measured to study the contact angle hysteresis (CAH) through the adding/removing liquid method at a room temperature of 10 °C. Table 2 shows that the fluorinated coating surfaces, CS#1-PFDTES and CS#2-PFDTES, produced CAH of 7.5 ± 2.6° and 7.2 ± 2.86°, which indicated that the Cassie–Baxter contact state was maintained during the test conditions.

It has been proven to be very difficult to maintain the Cassie–Baxter state on a surface exposed to environmental condensation, even for a superhydrophobic surface, especially in low-temperature and high-humidity environments. The condensate droplets tended to penetrate the surface texture and stuck to the subcooled surface, which led to the transition from the Cassie–Baxter state to the Wenzel contact model. Compared to the porous coating surfaces CS#1 with ~30 μm diameter particles, CS#2 coating surfaces yielded more stable water-repellence at low-temperature conditions, as shown in Figure 8a,b. Both for the porous coating surfaces with 500 nm and ~30 μm diameter, the fluorinated and slippery liquid-infused surfaces yielded a higher apparent contact angle than the non-treated porous coating surface. Smaller particle diameters produced higher porosity, more homogeneous surface roughness, and more trapped air cavities, which can contribute to maintaining the Cassie–Baxter contact model.

### 3.4. Anti-Icing Performance

**Figure 9:**
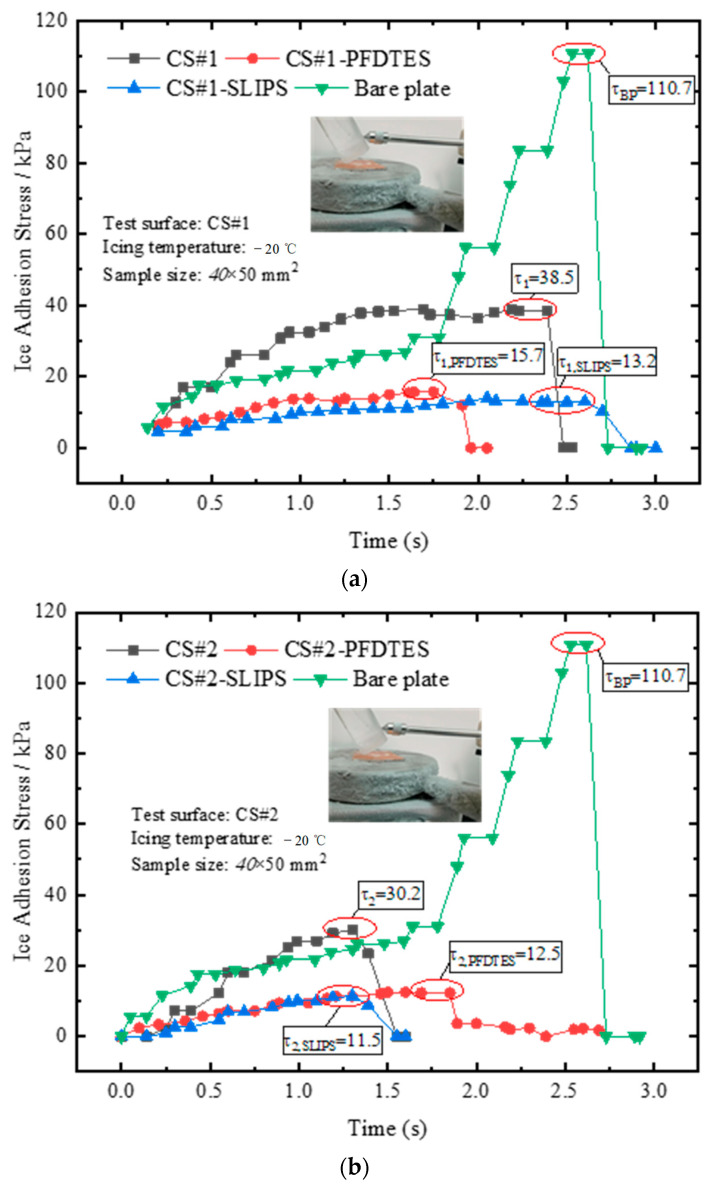
Results of ice adhesion stress measured by a pull-push dynamometer after the ice cylinder freezing for half an hour on (**a**) coating surface CS#1, CS#1-PFDTES, and CS#1-SLIPS, and (**b**) coating surface CS#2, CS#2-PFDTES, and CS#2-SLIPS, respectively.

Ice adhesion strength was the key parameter for evaluating the anti/deicing performance of various coating materials. The static icing experiments of the bare copper plate, CS#1 and CS#2 porous coating surfaces, and the corresponding fluorinated and slippery liquid-infused surfaces were performed on an isothermal cold plate at a temperature of −20 °C. The humidity of the local atmosphere was 45%, and an ice cylinder was prepared. Before placing the cylindrical ice on the plate, the bottom surface of the ice cylinder was wetted to accelerate the icing process. The ice adhesion stress was measured after icing on the cold plate for half an hour and recorded for all tested surfaces. The averaged ice adhesion stress of 10 icing and deicing circles was calculated for each surface, and the final results were presented in Figure 9.

The maximum ice adhesion stresses of the bare copper plate and non-treated coating porous surfaces CS#1 and CS#2 were 110.7 kPa, 38.5 kPa, and 30.2 kPa, respectively. This result indicated that the micro/sub-micro porous coating layer exhibited a significant decrease in ice adhesion strength, ascribed to the trapped air in the porous coating layer. The trapped air reduced the contact area and the positive pressure on the ice cylinder, decreasing the friction force between the ice cylinder and the substrate. Compared with the fluorinated coating surfaces, the ice adhesion stresses were only 15.7 kPa and 12.5 kPa for the CS#1-PFDTES and CS#2-PFDTES surfaces. The introduced chemical materials (PFDTES) replaced the water molecules bonding with the copper substrate, and the porous structure increased the bonding density, which prevented the formation of hydrogen bonds between the liquid layer and substrate. The polymer layer between the ice surface and the solid surface also increased the thermal contact resistance that prevented heat from flowing from the liquid phase to the substrate. Hejazi et al. [39] theoretically confirmed that the main parameter affecting droplet adhesion to a solid surface was CA hysteresis, while the receding CA and the porosity/roughness size were both important for anti/deicing application. Our test results also indicated that a surface with a higher receding contact angle and lower contact angle hysteresis could yield much lower ice adhesion strength.

For the slippery liquid-infused porous coating surfaces, CS#1-SLIPS and CS#2-SLIPS, the ice adhesion stresses were as extremely low as 13.2 kPa and 11.5 kPa. The simethicone with a viscosity of 20 mPa·s was infused in the porous layer, and an oil film formed on the rough surface. The oil film prevented the water droplet from direct contact with the substrate, forming an air-water-oil-copper contact system. The oil film also decreased the efficiency of heat transfer between the liquid phase and the copper substrate because the thermal resistance of the water-oil-solid contact surface was much higher than the water-solid contact system. In addition, the existence of the oil film isolated the oxhydryl bonding with the copper surface, which greatly reduced the ice adhesion force.

According to the test results, the surface roughness, chemical materials with low surface energy, icing temperature, and the thermal resistance between the substrate and ice layer were the key parameters that affected the anti/deicing characteristics of a porous coating surface. Based on the ice nucleation theory, the energy barrier for heterogeneous nucleation was much lower than the homogeneous nucleation, which can be expressed as ΔG_hete_ = ΔG_homo_ × *f = f* × *πσ_lv_r_c_*^2^(2 − 3cos *θ +* cos^3^ *θ*) indicating that the contact angle and the surface roughness were the most important parameters to prevent icing process. Where *f*, *σ_lv_*, and *r_c_* represented the surface roughness factor, liquid-vapor interfacial tension, and critical nucleus radius (~20 nm in Reference [15]). The nucleation rate, *J_ϕ_ = J*_0_ × *exp*(−ΔG_hete_*/k_b_T_s_*) was also influenced by the energy barrier and the icing temperature, which pointed out that the icing process will be much faster at low-temperature conditions. In this case, preventing heat transfer from liquid to the substrate will become more important to delay ice formation.

## 4. Conclusions

Two porous coated surfaces were fabricated by sintering micro and sub-micro copper powders on the plate substrate. The slippery liquid-infused surface was obtained by infusing simethicone into the porous layer, and surface superhydrophobicity was observed using the PFDTES material. The surface wettability and anti-icing characteristics were tested on a cold plate. The outcome of this experiment showed that coating the surface with sub-micro particles yielded better water repellency and lower ice adhesion strength compared with the micro-particle surface. The fluorinated porous coating surfaces produced the maximum contact angle (153 ± 2.64° and 155 ± 3.1°) and minimum contact angle hysteresis of ~7 ± 2.64°. The trapped air and the capillary force in the porous layer helped to maintain the Cassie–Baxter contact state, which contributed to reducing the ice adhesion force. The unordered micro/sub-micro interspace in the porous layer contributed to the balance of the atmospheric pressure. Both superhydrophobic and slippery liquid-infused porous coating surfaces exhibit a distinct decrease in ice adhesion strength. The porous layer can work as a metallic framework by introducing other nanomaterials to increase the thermal resistance between ice and substrate to survive in low temperature and high humidity conditions. The technology used to fabricate the porous coating is economical and practical and can be widely used for mass production. This work’s key findings can be further applied to the anti-icing of engine turbine blades and high-voltage transmission lines.

## Figures and Tables

**Table 1:** Results of energy dispersive spectrum analysis of CS#1-PFDTES and CS#2-PFDTES surfaces.

Element Type	Spectral Lines	Mass Fraction, % wt.		Atomic Ratio, %	
		CS#1-PFDTES	CS#2-PFDTES	CS#1-PFDTES	CS#2-PFDTES
C	K	7.93	7.81	30.36	30.31
O	K	0.75	0.50	2.15	1.47
F	K	0.75	0.49	1.83	1.21
Si	K	0.13	0.11	0.21	0.18
Cu	K	90.44	91.08	65.45	66.83
Sum.		100.00	100.00	100.00	100.00

**Table 2:** Wetting properties at coolant temperature and porous coating parameters of tested surfaces.

Sample Type	Wettability				Coating Layer Parameters	
	CA, deg.	ACA, deg.	RCA, deg.	CAH, deg.	D/µm	Thk./mm
Bare Cu plate	70.1	109.3	86.4	22.9	Bare surface	~0.1
CS#1	134.6	138.8	121.6	17.2	~30	~0.1
CS#1-PFDTES	153.4	156.2	148.7	7.5	~30	~0.1
CS#1-SLIPS	132.1	138.1	121.6	16.5	~30	~0.1
CS#2	142.3	147.1	136.3	10.8	~0.5	~0.1
CS#2-PFDTES	155.6	158.6	151.4	7.2	~0.5	~0.1
CS#2-SLIPS	144.1	151.2	137.8	13.4	~0.5	~0.1

## Data Availability

Not applicable.
